# Гормональные и генетические причины развития крипторхизма

**DOI:** 10.14341/probl13242

**Published:** 2023-11-12

**Authors:** Е. М. Орешкина, Н. В. Болотова, Т. Е. Пылаев, А. П. Аверьянов, Н. Ю. Райгородская

**Affiliations:** Саратовский государственный медицинский университет им. В.И. Разумовского; Саратовский государственный медицинский университет им. В.И. Разумовского; Саратовский государственный медицинский университет им. В.И. Разумовского; Саратовский государственный медицинский университет им. В.И. Разумовского; Саратовский государственный медицинский университет им. В.И. Разумовского

**Keywords:** крипторхизм, эмбриональная миграция яичка, генетические причины крипторхизма, клетки Лейдига и Сертоли, инсулиноподобный пептид 3, антимюллеров гормон, гипогонадотропный гипогонадизм

## Abstract

Крипторхизм — частая врожденная патология половой системы, определяемая с частотой 2–3% среди доношенных новорожденных мальчиков. На сегодняшний день выделены гены-кандидаты, участвующие в эмбриональной миграции яичка, однако данных об их роли в развитии крипторхизма недостаточно. Генетические причины крипторхизма удается установить в 5–7% случаев. В обзоре собраны данные о роли инсулиноподобного пептида 3, антимюллерова гормона, гонадотропных гормонов и андрогенов в эмбриональной миграции яичка. Проанализированы результаты оригинальных исследований частоты и характера мутаций гена INSL3 и его рецептора (RFXP2) при различных вариантах крипторхизма. Представлены данные о значении инсулиноподобного пептида 3 и антимюллерова гормона как маркеров нарушения функции яичек, ассоциированной с развитием крипторхизма. Показаны возможности диагностики врожденного гипогонадотропного гипогонадизма, ассоциированного с развитием крипторхизма. Анализ результатов современных исследований определяет необходимость гормонального и генетического обследования пациентов с изолированным крипторхизмом для выявления причин его развития и определения тактики ведения пациентов.

## Abstract (en)

Cryptorchidism is the most frequent congenital disorders of the reproductive system, is present in 2–3% of term newborn boys. Genes involved in embryonic testicular migration are known but their role in cryptorchidism development are not investigated enough. Genetical causes of cryptorchidism are identified in 5–7% of patients. The article contains data on the role of insulin-like peptide 3 and its receptor, anti-Müllerian hormone, gonadotropins, androgens in embryonic testicular migration. INSL3 and AMH are presented as markers of testicular dysfunction associated with cryptorchidism. Hypogonadotropic hypogonadism is also associated with cryptorchidism and can be diagnosed based on it. Results of modern investigations determine the necessary of hormonal and genetical examination of patients with isolated cryptorchidism to detect causes of cryptorchidism and manage of patients.

## ВВЕДЕНИЕ

Крипторхизм — частая врожденная патология половой системы, определяемая с частотой 2–3% среди доношенных новорожденных мальчиков. Этиология врожденного крипторхизма гетерогенна. Генетические причины крипторхизма плохо изучены и установлены у очень небольшого числа пациентов. Развитие крипторхизма часто обусловлено эндокринными нарушениями. Крипторхизм может быть проявлением хромосомной патологии и различных моногенных заболеваний. Двусторонний крипторхизм может являться ранним клиническим проявлением врожденного гипогонадотропного гипогонадизма либо отражать нарушение внутриутробного развития самого яичка. Однако в большинстве случаев пациенты с крипторхизмом наблюдаются детскими хирургами, и причины его развития остаются неверифицированными. Послеоперационное динамическое наблюдение не является обязательным, вследствие чего недостаточно данных о катамнезе пациентов с крипторхизмом.

Анализ данных современных исследований о генетических причинах и клиническом наблюдении пациентов с крипторхизмом необходим для поиска оптимального подхода к диагностике и наблюдению мальчиков с крипторхизмом.

Процесс внутриутробной миграции яичка реализуется под контролем генетических и гормональных факторов. Трансабдоминальная фаза — этап эмбриональной миграции от места первичной закладки яичка до внутреннего пахового кольца. Пахово-мошоночная фаза включает прохождение яичка через паховый канал, от его внутреннего кольца до дна мошонки [1][2]. Изучению генетического и гормонального контроля эмбриональной миграции яичка посвящено большое количество экспериментальных исследований, но клинические исследования о генетических причинах крипторхизма малочисленны и недостаточно информативны.

## ФИЗИОЛОГИЧЕСКАЯ РОЛЬ ИНСУЛИНОПОДОБНОГО ПЕПТИДА 3 (INSL3) В ЭМБРИОНАЛЬНОЙ МИГРАЦИИ ЯИЧЕК

В процессе трансабдоминальной миграции яичка ключевую роль играет инсулиноподобный пептид 3 (INSL3). INSL3 секретируется фетальными клетками Лейдига с 10-й по 15-ю неделю внутриутробного развития под действием хорионического гонадотропина [2][3]. INSL3 отражает степень дифференцировки и функционального состояния эмбриональных клеток Лейдига [4][5]. Bay K.V. и соавт. выявили положительные корреляции INSL3 с уровнем лютеинизирующего гормона (ЛГ) и тестостерона у здоровых мальчиков в возрасте 3 мес и обнаружили снижение концентрации INSL3 при завершении периода мини-пубертата наряду с физиологическим снижением секреции гонадотропинов [4].

Ключевым моментом трансабдоминальной фазы миграции яичка является дифференцировка губернакулюм и закрепление яичка в паховом канале, что происходит под контролем INSL3 и его рецептора RFXP2 [6][7]. При исследовании образцов пуповинной крови 12 плодов наблюдалась тенденция к увеличению концентрации INSL3 в сыворотке плода с 15 до 20 нед гестации [8]. Пик максимального внутриутробного повышения INSL3 приходился на 15–18 нед гестации, что совпадает с дифференцировкой губернакулюм и окончанием абдоминальной фазы опущения яичек. Авторы предполагают, что увеличение продукции INSL3 после 15-й недели свидетельствует не только об участии данного фактора в трансабдоминальной фазе опущения яичек, но и в пахово-мошоночной, за счет расширения пахового канала.

Гипотеза об участии INSL3 и его рецептора во второй фазе миграции яичек подтверждается в экспериментальном исследовании на животных [9]. У мышей с паховым крипторхизмом и инактивацией рецептора ЛГ введение тестостерона вызывало активацию экспрессии мРНК RXFP2 в губернакулюм, а введение INSL3 активировало дифференцировку ее клеток, что в совокупности способствовало опущению яичек в мошонку. Лечение тестостероном в отсутствие INSL3 не приводило к дифференцировке клеток губернакулюм, а INSL3 значительно усиливал клеточное деление только в присутствии тестостерона. Введение антагониста INSL3 приводило к подавлению сигнального пути RXFP2 и снижало INSL3-индуцированную пролиферацию клеток губернакулюм. Эти данные свидетельствуют о том, что андрогенная регуляция пахово-мошоночного опускания яичка опосредована активацией экспрессии RXFP2 в губернакулюм.

Участие INSL3 во второй фазе миграции яичек изучалось в исследованиях с участием новорожденных [10]. Уровень INSL3 у 43 детей с крипторхизмом был снижен и составил 225,7±19,3 нг/л в сравнении с контрольной группой здоровых новорожденных — 271,4±18,4 нг/л (P=0,031). Концентрации тестостерона не отличались между исследуемой и контрольной группами, 0,47±0,45 нмоль/л и 0,41±0,31 нмоль/л соответственно (P=0,7).

Понимание фундаментальной роли INSL3 в опущении яичек побудило к активному поиску мутаций или полиморфизмов в гене INSL3 и его рецепторе у пациентов с идиопатическим крипторхизмом. По данным исследований, мутации генов INSL3 и RFXP2 (LGR8) в гомозиготном состоянии были выявлены у небольшого числа пациентов с крипторхизмом. В исследовании C. Foresta, A. Ferlin приняли участие 135 взрослых мужчин в возрасте 33,0±2,8 года, которые перенесли оперативное лечение по поводу одностороннего (n=55) и двустороннего (n=72) крипторхизма. Мутации генов INSL3 и RFXP2 были найдены у 7,4% пациентов. Нуклеотидные замены L9L, A24G, A60T и L42L были выявлены как у мужчин с крипторхизмом, так и у контрольной группы, которые были расценены как полиморфизмы. Две мутации P93L, одна мутация R102C и три мутации R102H были обнаружены в гетерозиготном состоянии при двустороннем крипторхизме у 5,4% мужчин и при одностороннем крипторхизме у 4,2% пациентов. Эти мутации не были обнаружены в группе контроля, на основании чего авторы предполагают, что они могут быть рассмотрены как патогенные [11].

В исследовании A. Ferlin и соавт. с участием 540 пациентов с различными вариантами крипторхизма было выявлено три полиморфизма: A24G, A60T и V43L. Мутации R102C, R102H, P93L и W69R были обнаружены в гетерозиготном состоянии в 2,2% случаев. Эти нуклеотидные замены не были обнаружены в контрольной группе. У пациентов с мутациями R102H и P93L отмечалась олигозооспермия различной степени выраженности. Пациенты с мутацией W69R страдали бесплодием, у одного из них была выявлена семинома [12].

До недавнего времени миссенс-мутация Т222P в гене RXFP2 (LGR8) считалась наиболее распространенной и вероятной причиной трансабдоминальной задержки яичка. Несколько исследований были посвящены изучению клинической значимости этой замены, однако данные о патогенности Т222P оказались противоречивыми. В исследовании C. Foresta, A. Ferlin и соавт. гетерозиготная мутация T222P была выявлена у 1,8% пациентов с двусторонним крипторхизмом и 2,8% мужчин с односторонним крипторхизмом [11]. В исследовании A. Ferlin, M. Simonato и соавт. с участием 87 мужчин мутация T222P была выявлена путем прямого секвенирования гена RFXP2 у 4,6% пациентов с различными вариантами крипторхизма [13]. Данная мутация также была описана у ребенка с двусторонним крипторхизмом в сочетании с члено-мошоночной гипоспадией и микропенией при кариотипе 46, ХY [14]. В исследовании A. Ferlin (2006 г.) среди 303 детей с крипторхизмом мутация T222P была выявлена в 5 случаях, и все они отмечены в группе мальчиков с двусторонним крипторхизмом. Частота данной мутации у детей составила 4,2%. В контрольной группе этой мутации обнаружено не было [15][16].

Параллельно группа ученых из Италии, Испании и Египта провели скрининг миссенс-мутации T222P гена RXFP2 у 187 испанских и у 199 итальянских мужчин с различными формами крипторхизма в анамнезе. В испанской когорте вариант T222P присутствовал с одинаковой частотой как у пациентов с крипторхизмом — 1,6%, так и в группе контроля — 1,8%. В группе итальянцев данный вариант встречался значительно чаще у мужчин с крипторхизмом — 4,5%, чем в контрольной группе — 1,4%. На основании полученных результатов авторы предполагают, что гетерозиготная замена Т222P не является патогенным вариантом и обозначают необходимость расширения поиска причин крипторхизма [17]. В генетической базе данных ClinVar мутация присутствует как вариант неопределенной клинической значимости. Остается открытым вопрос, является ли патогенной мутация T222P гена RFXP2 в гомозиготном состоянии.

Особый интерес с точки зрения клинической значимости мутаций представляют описания семейных случаев крипторхизма. Патогенный вариант RFXP2 с аутосомно-рецессивным типом наследования был описан в семейном случае у четырех мальчиков с изолированным двусторонним крипторхизмом. Было проведено полное секвенирование экзома у четырех мальчиков и их родителей. Геномный анализ выявил гомозиготный миссенс-вариант гена RXFP2 (c.1496G>A.p.Gly499Glu) у всех пациентов и гетерозиготный вариант у обоих родителей. Других вариантов, которые могли бы являться причиной крипторхизма, обнаружено не было [18].

Таким образом, общая частота встречаемости мутаций INSL3 и RFXP2, по данным клинических исследований, составляет от 2,3 до 9,2%. Наиболее вероятные патогенные варианты мутаций INSL3 встречаются практически с одинаковой частотой среди пациентов с односторонним и двусторонним крипторхизмом, тогда как мутации RXFP2 чаще выявлены при двустороннем крипторхизме. В большинстве исследований не описан уровень расположения гонад у исследуемых пациентов, в связи с чем сложно сопоставить выявленные мутации с клиническими вариантами крипторхизма, невозможно оценить распространенность мутаций среди паховых и абдоминальных форм, что требует дальнейшего изучения.

## РОЛЬ АМГ В РАЗВИТИИ КРИПТОРХИЗМА

Антимюллеров гормон (АМГ) является еще одним фактором, контролирующим трансабдоминальную фазу миграции яичка. В раннем эмбриональном периоде АМГ секретируется незрелыми клетками Сертоли и не зависит от контроля фолликулостимулирующего гормона (ФСГ). Под воздействием АМГ мюллеровы протоки регрессируют у плода мужского пола на 8–9-й неделе внутриутробного развития, препятствуя развитию матки, маточных труб и проксимальной части влагалища [19]. При мутациях в гене AMH и его рецептора у плодов мужского пола развивается синдром персистенции мюллеровых протоков (СПМП), который характеризуется абдоминальным крипторхизмом и наличием производных мюллеровых протоков [20]. Абдоминальная задержка яичка при СПМП возникает в результате механического препятствия. Гонада остается прикрепленной к фаллопиевой трубе, которая препятствует ее опусканию [21]. В экспериментальных исследованиях было показано, что у животных с инактивацией гена рецептора AMH отмечается нормальное опущение яичек в мошонку [22]. Авторами были обследованы 11 самцов мышей с гетерозиготными мутациями рецептора AMH и 12 мышей с гомозиготными мутациями. Мюллеровы протоки присутствовали у гомозигот, частично присутствовали у гетерозиготных самцов и отсутствовали у контрольной группы животных. Кремастерная мышца была значительно хуже развита к моменту рождения у гомозиготных животных, чем у гетерозигот (Р<0,01) и у контрольной группы (P<0,001). Несмотря на наблюдаемую разницу в развитии кремастерных мышц, у мышей с мутациями рецептора AMH развитие губернакулюм было нормальным и у всех самцов яички были опущены в мошонку. Таким образом, авторы делают вывод, что у людей с синдромом персистирующих мюллеровых протоков крипторхизм развивается в результате механических препятствий для миграции яичка в процессе абдоминальной фазы.

Концентрация сывороточного АМГ также отражает функцию клеток Сертоли. В исследовании F. Tüttelmann и соавт. изучали взаимосвязь сывороточного АМГ с уровнем гонадотропных гормонов и половых стероидов, объемом гонад, концентрацией сперматозоидов у пациентов с олигоспермией, крипторхизмом и у здоровых мужчин в возрасте от 20 до 53 лет. У пациентов с крипторхизмом были выявлены сильные обратные корреляции АМГ с уровнем ФСГ и прямые корреляции АМГ с объемом гонад и концентрацией сперматозоидов в эякуляте, чего не отмечалось в других группах [23]. Корреляций между АМГ и ЛГ или тестостероном не выявлено. Авторы делают вывод, что АМГ можно считать маркером числа, функции и созревания клеток Сертоли именно у пациентов с крипторхизмом. При этом его определение в рутинной клинической практике нецелесообразно, уровень АМГ не отражает степень пролиферации клеток Сертоли у здоровых мужчин в отличие от препубертатных мальчиков [19][23]. Действительно, уровень АМГ высок у плода, затем его концентрация увеличивается в несколько раз в период мини-пубертата под воздействием ФСГ, что обеспечивает пролиферацию незрелых клеток Сертоли [24].

В исследовании Grinspon R.P. и соавт. описаны изменения уровня АМГ у препубертатных мальчиков с крипторхизмом [25]. Проанализированы результаты исследования АМГ 310 препубертатных мальчиков (Tanner 1 и объем гонад ≤3 мл): 186 с двусторонним крипторхизмом и 124 с односторонним крипторхизмом. Уровень АМГ мальчиков с крипторхизмом значимо не отличался от контроля в возрастной группе 1–6 мес, но в группах от 6 мес до 1,9 года и от 2 до 8,9 года был существенно ниже. Концентрация АМГ оказалась ниже 3 перцентиля у 22,6% мальчиков с двусторонним и у 8,1% мальчиков с односторонним крипторхизмом. На основании исследования большой когорты пациентов авторы делают вывод о тестикулярной дисфункции большого числа пациентов с крипторхизмом. Снижение секреции АМГ, главным образом при билатеральном крипторхизме, — результат снижения числа клеток Сертоли в неопущенных яичках и/или нарушения их функции.

## РОЛЬ АНДРОГЕНОВ В ОПУЩЕНИИ ЯИЧЕК

Андрогены не играют значимой роли в первой фазе опускания яичек. При нарушениях биосинтеза или действия андрогенов гонады чаще всего преодолевают абдоминальную фазу и определяются по ходу паховых каналов [26][27]. Основная роль андрогенов — в обеспечении прохождения яичка через паховый канал и закрепление его положения в мошонке. В экспериментальных исследованиях на грызунах было показано, что андрогены влияют на генитофеморальный нерв (GFN), взаимодействуя с его медиатором CGRP (calcitonin gene-related peptide), которые регулируют пролиферацию и подвижность губернакулюм [28]. В американском исследовании E.M. Kaftanovskaya и соавт. изучались механизмы передачи сигнала AR (андрогенового рецептора) в губернакулюм в процессе пахово-мошоночной фазы миграции яичка. Исследователи установили, что инактивация AR в клетках губернакулюм у экспериментальных животных приводит к развитию пахового крипторхизма, нарушению развития кремастерной мышцы и инфертильности. При гистологическом исследовании гонад обнаружено аномальное строение семенных канальцев, снижение количества сперматозоидов в результате вакуолизации клеток Сертоли [29]. Авторы сделали вывод, что именно губернакулюм является органом-мишенью для андрогенов в процессе второй фазы миграции яичек, а также подтвердили связь крипторхизма и инфертильности.

Поскольку андрогены играют решающую роль в пахово-мошоночной фазе опущения яичка, многие заболевания и синдромы, связанные с гипогонадизмом или снижением действия андрогенов, могут проявляться крипторхизмом. Мутации в гене AR вызывают синдром нечувствительности к андрогенам разной степени, от более тяжелых форм с полным женским фенотипом до частичной нечувствительности с микропенией, гипоспадией и крипторхизмом или самых легких форм с нарушением только сперматогенеза [30].

В некоторых исследованиях изучалась связь между длиной повторов нуклеиновых кислот CAG и GGN в 1 экзоне гена AR и крипторхизмом. В метаанализе Q. Wang были выделены группы с односторонним и двусторонним крипторхизмом, и область повторов CAG была значительно длиннее у пациентов с двусторонним крипторхизмом (р<0,05). Также было выявлено, что длина повторов GGN была значительно выше у пациентов с крипторхизмом по сравнению с контрольной группой. Авторы делают выводы, что полиморфизм повторов AR CAG и GGN может нарушать функцию AR и таким образом влиять на процесс опущения яичек [31]. Количество повторов CAG в гене AR также было изучено в недавнем исследовании D.A. Landero-Huerta с участием 62 мексиканских пациентов с изолированным паховым крипторхизмом, двустороннюю форму имели 30/62 мужчин. При анализе длины области CAG у пациентов с крипторхизмом было выявлено 25,03±2,58 повтора в сравнении с 22,72±3,1 в контрольной группе (р≤0,0001). Количество повторов CAG в гене AR преобладало у пациентов с крипторхизмом и подтверждало влияние полиморфизма гена AR на развитие крипторхизма [32].

Андрогензависимая фаза миграции яичка происходит под контролем ЛГ гипофиза плода в период с 27-й по 35-ю недели внутриутробного развития. Отсутствие или снижение концентрации ЛГ в данный период или поражение ЛГ-рецептора способствует развитию крипторхизма [33]. В экспериментальных исследованиях инактивация ЛГ-рецепторов у животных вызывала гипоплазию клеток Лейдига, снижение выработки INSL3 и тестостерона. Гистологическое исследование показало нарушение дифференцировки губернакулюм и кремастерных мышц, что способствовало формированию двусторонней задержки яичка [9].

## ГЕНЫ-КАНДИДАТЫ, УЧАСТВУЮЩИЕ В РАЗВИТИИ КРИПТОРХИЗМА

Результаты исследований на животных и небольшие клинические исследования позволили выделить другие потенциальные гены-кандидаты, участвующие в развитии крипторхизма. Есть данные о том, что ген опухоли Вильмса 1 (WT1) участвует в дифференцировке губернакулюм и влияет на опущение яичек, а также может регулировать экспрессию гена AR [34][35]. В исследовании с участием экспериментальных животных при иммуногистохимическом анализе была обнаружена выраженная экспрессия WT1 в клетках Сертоли и губернакулюм. У 40% мышей с делецией гена WT1 был выявлен левосторонний крипторхизм [35].

Еще одним геном-кандидатом, ассоциированным с развитием крипторхизма, является HOXA10. Ген HOXA10 расположен на 7 хромосоме и играет роль в развитии губернакулюм во внутриутробном периоде [36]. При анализе гена HOXA10 в группе из 98 пациентов с различными формами крипторхизма была выявлена мутация N27K у мальчика с односторонним крипторхизмом. Данного варианта не было обнаружено в контрольной группе. Авторы предполагают, что мутации в гене HOXA10 участвуют в патогенезе крипторхизма, но не являются частой причиной [37].

F. Rodriguez и соавт. предположили, что гены Ras/MARK pathwayучаствуют в развитии изолированного крипторхизма. В исследовании приняли участие 59 мальчиков в возрасте 1–15 лет с изолированным крипторхизмом. У 5% мальчиков с односторонним и двусторонним крипторхизмом методом сравнительной геномной гибридизации были обнаружены микродупликации гена RAF1. Уровни ЛГ, ФСГ и тестостерона в сыворотке обследованных пациентов соответствовали возрастным нормам, но отмечалось значительное снижение концентрации ингибина В [38]. Участие гена RAF1в развитии крипторхизма было показано в исследовании N.O. Hadziselimovic, C. Geyter и соавт. Авторами проанализированы результаты биопсии яичек 18 мальчиков с различными формами изолированного крипторхизма. В биоптатах неопущенных яичек выявлено снижение экспрессии генов рецептора фактора роста фибробластов (FGFR1), SOS1 и RAF1. Авторы предполагают, что нарушение экспрессии генов FGFR1, SOS1 и RAF1 может являться причиной развития крипторхизма [39].

## КРИПТОРХИЗМ И ГИПОГОНАДОТРОПНЫЙ ГИПОГОНАДИЗМ

Двусторонний паховый крипторхизм или крипторхизм в сочетании с микропенией может являться манифестным симптомом врожденного гипогонадотропного гипогонадизма (гипоГГ) [40]. По данным исследований, частота крипторхизма у пациентов с гипоГГ составляет 40–69,6% [41][42]. При мутациях гена KAL-1 двусторонний крипторхизм встречается у 67% пациентов, при мутациях гена FGFR1/KAL-2 — у 20% [43]. При гипоГГ, обусловленном мутацией генов PROK2 и PROKR2, были установлены корреляции между тяжестью мутации и выраженностью клинической картины. При моноаллельных мутациях у пациентов реже встречались крипторхизм и микропения, отмечались больший объем яичек и более высокие уровни в сыворотке крови ЛГ, ФСГ и тестостерона, чем при биаллельных [27].

В исследовании E.M. Laitinen, J. Tommiska (2011) изучалась роль генов FGFR1, PROK2, PROKR2, TAC3 или TACR3, лежащих в основе гипоГГ, в этиологии изолированного крипторхизма. В исследовании приняли участие 54 пациента с различными формами крипторхизма. Мутаций данных генов у пациентов выявлено не было. Гетерозиготные мутации гена GNRHR были выявлены у 2 пациентов с односторонним крипторхизмом, но они также присутствовали у контрольной группы мужчин (p=0,62). Авторы делают заключение, что изолированный крипторхизм не связан с патологией генов, лежащих в основе гипоГГ [44].

Патогенетической основой крипторхизма при врожденном гипоГГ является отсутствие внутриутробной и постнатальной выработки собственного ЛГ и тестостерона [45]. Определение уровней гонадотропных и половых гормонов у пациентов с гипоГГ в период мини-пубертата дает возможность ранней диагностики данного состояния и позволяет планировать инициацию пубертата в физиологические сроки, предотвращая возможную задержку полового созревания [46].

## МИНИ-ПУБЕРТАТ И КРИПТОРХИЗМ

Ряд исследований посвящен изучению особенностей течения периода мини-пубертата у мальчиков с изолированным крипторхизмом. В исследование K. Bay и соавт. были включены 62 доношенных новорожденных мальчика с крипторхизмом. Уровень INSL3, тестостерона и гонадотропных гормонов определяли в пуповинной крови сразу после рождения и в сыворотке крови в возрасте 2,5–4,5 мес. Мальчики с крипторхизмом имели сниженный уровень INSL3 пуповинной крови (р=0,001) в сравнении со здоровыми детьми. В возрасте 3 мес уровень INSL3 существенно не отличался, однако отмечалось значимое повышение соотношения ЛГ/INSL3 по сравнению с группой контроля (р=0,036). Выявленные у здоровых детей значимые положительные корреляции между INSL3 и ЛГ, тестостероном не наблюдались у мальчиков с крипторхизмом, что свидетельствовало о дисфункции клеток Лейдига. На основании полученных результатов авторы делают вывод, что INSL3, определяемый в период мини-пубертата, может служить маркером наличия функционирующей тестикулярной ткани и имеет клиническое значение при обследовании пациентов с нарушением формирования гонад [4].

В исследовании в Финляндии и Дании c участием 388 финских и 433 датских мальчиков с различными формами крипторхизма в возрасте 2,5–3,5 мес продемонстрированы более высокие уровни ЛГ (P<0,028), соотношений ЛГ/тестостерон (P<0,008) и ЛГ/свободный тестостерон (P<0,031), но одинаковые уровни тестостерона у мальчиков с крипторхизмом по сравнению с контрольной группой. Авторы предполагают, что уровень ЛГ повышается в период мини-пубертата компенсаторно, для поддержания нормального уровня тестостерона, и свидетельствует о нарушении функции клеток Лейдига. При анализе результатов также выявлены повышение концентрации сывороточного ФСГ (P<0,0001) и низкие показатели ингибина В (P<0,015) у мальчиков с крипторхизмом в период мини-пубертата. Полученные данные могут свидетельствовать о нарушении функции клеток Сертоли [47]. В отличие от результатов финского и датского исследований, у мальчиков в возрасте от 1 до 6 мес в Нидерландах и США не выявлено существенных различий в уровнях ФСГ, ингибина В или АМГ в сыворотке крови между детьми с крипторхизмом и контрольной группой [48][49].

Интерпретация вышеизложенных результатов затруднена из-за различий в возрасте при проведении исследований и отсутствия распределения пациентов в зависимости от уровня расположения яичек. В исследовании 2014 г. нами было показано, что пациенты с паховым и абдоминальным крипторхизмом имеют существенные различия клинико-гормональных показателей в период мини-пубертата [50]. В исследование вошли мальчики в возрасте 1–3 мес с паховым крипторхизмом (n=32) и абдоминальной задержкой яичка (n=10). Концентрации ЛГ и ФСГ в сыворотке крови у пациентов с паховым крипторхизмом были выше, чем у здоровых мальчиков. При этом уровни тестостерона и ингибина B практически соответствовали показателям здоровых детей. В группе мальчиков с абдоминальным крипторхизмом выявлено повышение концентрации ЛГ в сочетании со снижением уровня тестостерона у 50%, высокие значения ФСГ с одновременным снижением сывороточной концентрации ингибина B относительно группы контроля — у 80% мальчиков. Таким образом, установлены существенные различия клинико-гормональных показателей мальчиков с крипторхизмом в период мини-пубертата в зависимости от уровня расположения яичка.

## ЛЕЧЕНИЕ КРИПТОРХИЗМА

Лечение пациентов с крипторхизмом на сегодняшний день остается нерешенной проблемой. По рекомендациям Европейской ассоциации урологов, лечение крипторхизма следует начинать в возрасте 6–12 мес, не позднее 18 мес [51]. Это связано с результатами ряда исследований. При гистологическом исследовании паховых тестикул было выявлено прогрессирующее уменьшение количества герминативных клеток у детей старше 1–2 лет [52]. В исследовании C. Kollin, J.B. Stukenborg при гистологии 213 абдоминальных и паховых яичек количество герминативных клеток и клеток Сертоли, а также объем яичка в возрасте 9 мес были значительно больше, чем в 3 года. Объем яичка коррелировал с количеством герминативных клеток и клеток Сертоли в любом возрасте. Авторы делают следующие выводы: чем дольше яички находятся во внутрибрюшном положении, тем меньшее количество герминативных клеток сохраняется. Дооперационное положение яичек имеет меньшее значение, если операция выполняется в ранние сроки [53].

Предпочтительным методом лечения крипторхизма на сегодняшний день является орхиопексия. По рекомендациям Европейской и Американской ассоциаций урологов, не рекомендуется гормональное лечение для опущения яичек у пациентов с крипторхизмом, что связано с низкой эффективностью гормональной терапии и отсутствием долгосрочных результатов исследований [51][54]. По результатам исследований, направленных на сравнение хирургической и гормональной тактики лечения, орхиопексия была успешна у 95% пациентов. При терапии хорионическим гонадотропином человека (ХГЧ) и Гн-РГ опущение яичек в мошонку отмечалось только в 15% случаев [55][56]. В недавнем метаанализе Y. Wei, Y. Wang 2018 г. по результатам семи рандомизированных контролируемых исследований оценивалась эффективность лечения ХГЧ по сравнению с ГнРГ и плацебо. Анализ этих исследований показал, что лечение ХГЧ эффективно не больше, чем плацебо, и не было никаких существенных различий в эффективности лечения ХГЧ по сравнению с лечением ГнРГ при двустороннем (P=0,76) и при одностороннем крипторхизме (P=0,61) [57].

Некоторые исследования продемонстрировали пагубные последствия для жизнеспособности герминативных клеток яичка при использовании ХГЧ-терапии, нарушение сперматогенеза и уменьшение объема тестикул, на основании чего авторы сделали вывод о выборе орхиопексии как предпочтительного метода лечения при идиопатическом крипторхизме [51][54][58].

Таким образом, многочисленные исследования показали неэффективность гормональной терапии с целью опущения яичек при крипторхизме. Исключение составляют случаи, когда крипторхизм является одним из проявлений гипогонадотропного гипогонадизма.

C. Bouvattier и соавт. подчеркивают целесообразность терапии гонадотропинами у пациентов с крипторхизмом при врожденном гипоГГ для снижения необходимости хирургического лечения и нормализации уровня тестостерона, АМГ, ингибина В. Гормональное лечение крипторхизма у мальчиков с гипоГГ возможно в первые месяцы жизни — в период мини-пубертата [42].

В исследовании D.T. Papadimitriou, D. Chrysis был описан положительный эффект терапии гонадотропинами у 10 новорожденных мальчиков с гипоГГ в возрасте 3,5 мес (1,9–7,8). У всех пациентов отмечались двусторонний крипторхизм и микропенис ≤2 см. В ходе лечения длина полового члена увеличилась с медианы 2,0 до 3,8 см, со значительным повышением сывороточных уровней тестостерона, ингибина В и АМГ. В ходе терапии оба яичка опустились в мошонку у 8 пациентов. У 2 пациентов одно из яичек поднялось в паховый канал, в связи с чем было проведено хирургическое лечение до 1 года [59]. Терапия комбинацией препаратов рФСГ и тестостерона не оказала подобного эффекта на опущение яичек. Всем детям была проведена двусторонняя орхиопексия в возрасте 2,0±0,7 года. D.S. Swee, R. Quinton на основании вышеописанных результатов было высказано предположение о том, что экзогенная терапия тестостероном, в отличие от терапии рЛГ у детей с гипоГГ, не способна приводить к необходимой концентрации инстратестикулярных андрогенов и уровня INSL3, что необходимо для опущения и закрепления яичек в мошонке [60].

## ЗАКЛЮЧЕНИЕ

Анализ данных литературы показал сложные и разнообразные патофизиологические механизмы тестикулярной ретенции. Генетические причины крипторхизма мало изучены, что связано с многообразием этиологических факторов. Нами проанализированы результаты нескольких когортных исследований, направленных на верификацию причин крипторхизма, по результатам которых выявить генетические поломки, влияющие на процесс внутриутробной миграции яичка, удавалось в 5–7% случаев. В большинстве случаев результаты этих исследований были получены на разнородных когортах пациентов, включающих односторонние и двусторонние формы, паховую и абдоминальную задержку, что создавало сложности в оценке результатов.

До настоящего времени нет четкого алгоритма ведения пациентов с различными формами крипторхизма, подразумевающего междисциплинарный подход и включение эндокринологического обследования в программу наблюдения мальчиков с крипторхизмом. Это определяет необходимость дальнейших исследований.

## ДОПОЛНИТЕЛЬНАЯ ИНФОРМАЦИЯ

Источники финансирования. Работа выполнена в рамках проекта Перспективного научного исследования «Ранняя молекулярная верификация патологии полового развития в период мини-пубертата» Саратовского государственного медицинского университета им. В.И. Разумовского.

Конфликт интересов. Авторы декларируют отсутствие явных и потенциальных конфликтов интересов, связанных с публикацией настоящей статьи.

Участие авторов. Орешкина Е.М. — поиск и анализ литературы, написание основного текста статьи; Болотова Н.В. — поиск и анализ литературы, написание актуальности и заключения, редактирование текста статьи; Пылаев Т.Е. — поиск и анализ литературы, редактирование текста статьи; Аверьянов А.П. — поиск и анализ литературы, редактирование текста статьи; Райгородская Н.Ю. — поиск и анализ литературы, планирование и дизайн статьи, редактирование текста. Все авторы одобрили финальную версию статьи перед публикацией, выразили согласие нести ответственность за все аспекты работы, подразумевающую надлежащее изучение и решение вопросов, связанных с точностью или добросовестностью любой части работы.
